# Méningiome chordoïde extra-axiale: à propos d’un cas et revue de la littérature

**DOI:** 10.11604/pamj.2023.44.174.7539

**Published:** 2023-04-13

**Authors:** Khalid Skounti, Mohamed Mounadi, Jihed Mortada

**Affiliations:** 1 Service de Neurochirurgie, Hôpital Pasteur, Colmar, France

**Keywords:** Méningiome chordoïde, maladie de Castelman, MIB 1, cas clinique, Chordoid meningioma, Castelman’s disease, MIB 1, case report

## Abstract

Le méningiome chordoïde est inscrit selon la dernière classification des tumeurs cérébrales en classe II des méningiomes avec les méningiomes à grandes cellules. C'est une tumeur rare souvent accompagnée de symptômes systémiques décrites par Castelman. Nous présentons un cas de méningiome chordoïde découverte fortuitement chez une patiente de 45 ans suite à un accident de la voie publique, la tomodensitométrie cérébrale réalisée montre une lésion ptérionale gauche intra-diploïque isodense avec lyse osseuse, qui se rehausse de façon homogène après injection de produit de contraste, imagerie par résonnance magnétique montre une lésion hypointense en T1 et spontanément hyperintense en T2/FLAIR, et se rehausse très fortement après injection de gadolinium. Une résection complète de la tumeur a été réalisée. Le diagnostic histologique était de méningiome chordoïde en se basant sur l´étude immuno-histochimique.

## Abstract (en)

Chordoid meningioma is classified in the latest classification of brain tumours as a grade II meningioma, along with large cell meningiomas. It is a rare tumour often associated with systemic symptoms, as described by Castelman. He described a case of chordoid meningioma discovered incidentally in a 45-year-old female patient following road accident. Brain CT scan showed left isodense intradiploid pterional lesion with bone lysis, which was homogeneously enhanced after injection of contrast medium. Magnetic resonance imaging showed T1-hypointense and spontaneously T2 FLAIR hyperintense lesion, which was very strongly enhanced after injection of Gadolinium. Complete resection of the tumour was performed. Histological and immunohistochemical examination showed chordoid meningioma.

## Introduction

Les méningiomes chordoïdes est un rare sous-type de méningiomes parfois associé à la maladie de Castelman [1], il représente entre 0,5 et 1% de tous les méningiomes [2-4], à cause de son haut potentiel de récidive, elle a était classé parmi les méningiomes de grade 2 selon la dernière relecture de la classification des tumeurs du système nerveux établie par l´organisation mondiale de la santé [5]. Le but de ce travail est d´étudier les caractéristiques cliniques et biologiques de cette entité et de discuter les principaux diagnostics différentiels.

## Patient et observation

**Information de la patiente:** une femme âgée de 45 ans, sans antécédents médicaux ou chirurgicaux notables, qui a été victime d'un traumatisme crânien bénin suite à un accident de la voie publique pour lequel elle a consulté aux urgences.

**Résultats cliniques:** l'examen clinique était normal, notamment pas de troubles de conscience ni de signes méningés, ni de troubles de la sensibilité ou de la motricité. Le bilan biologique retrouve une anémie hypochrome microcytaire avec un taux d´hémoglobine à 8,1 g/dl mais sans syndrome inflammatoire.

**Figure 1:**
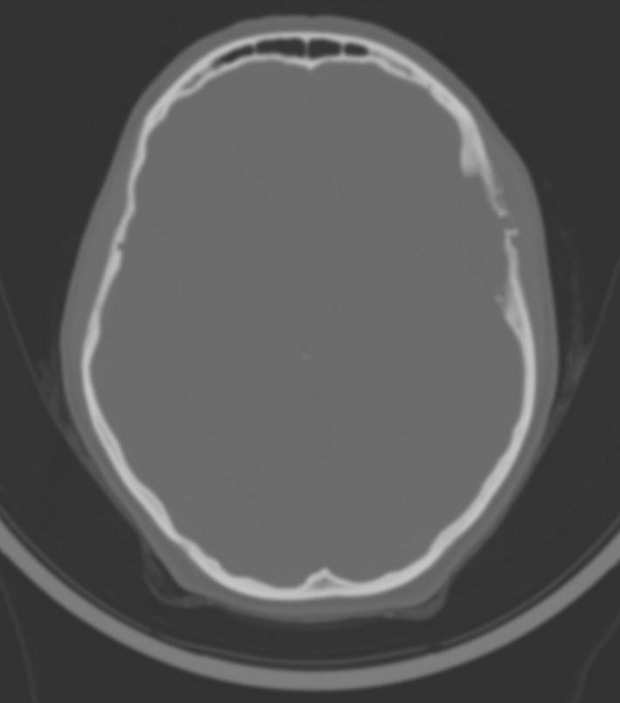
coupe axiale scanographique

**Figure 2:**
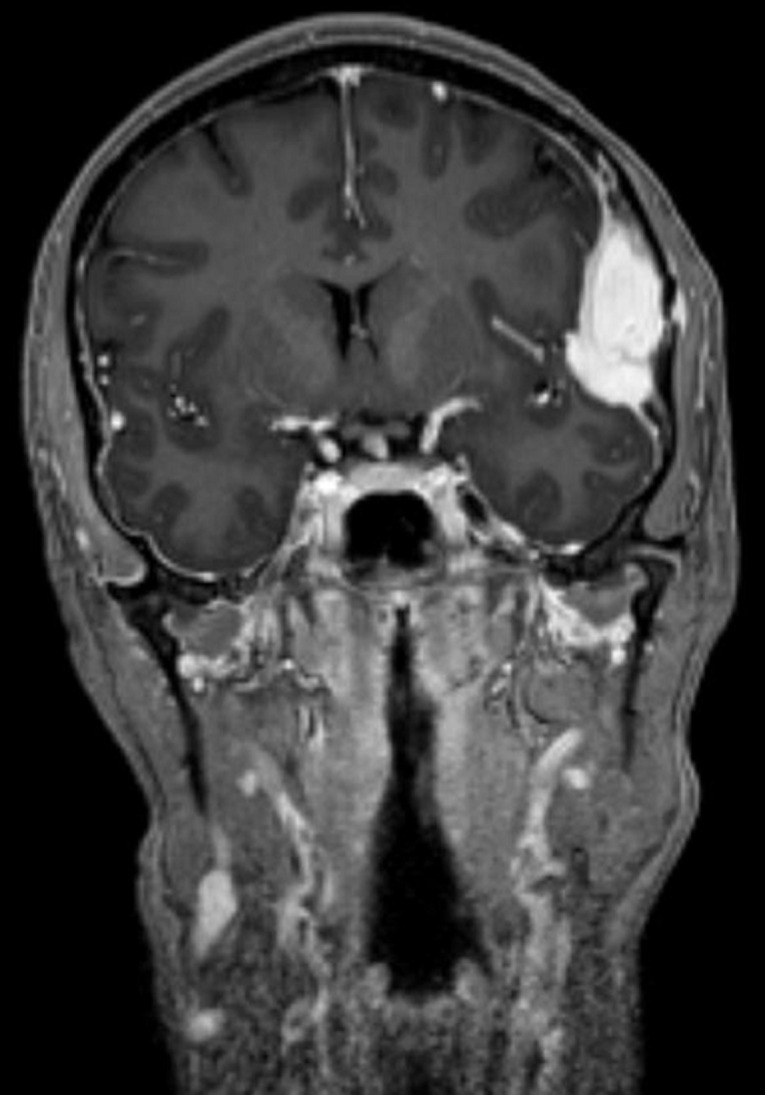
IRM coupe coronale préopératoire

**Figure 3:**
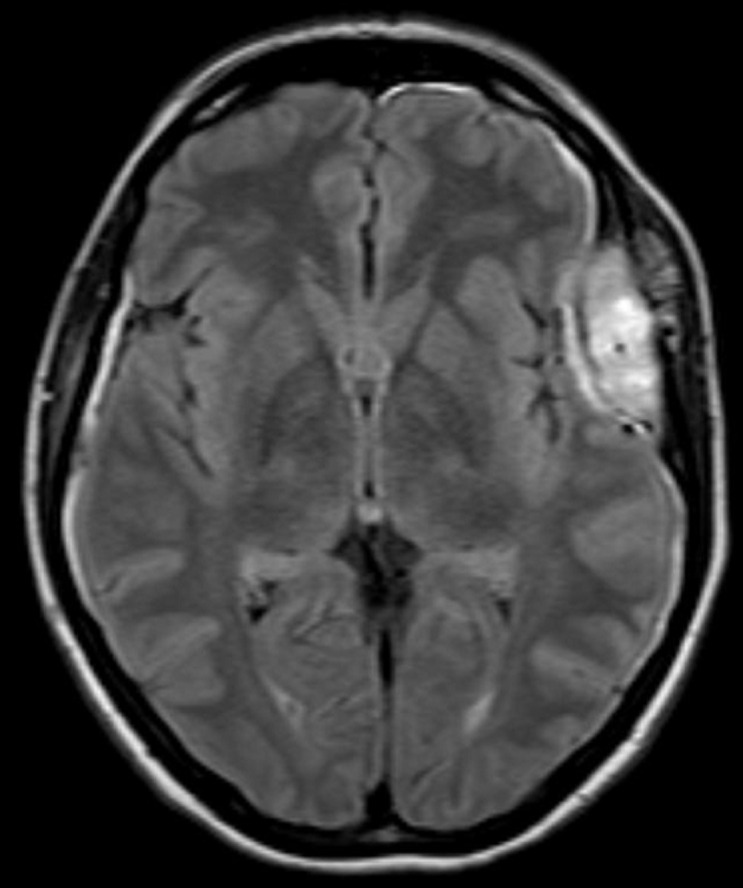
IRM coupe axiale pré-opératoire

**Démarche diagnostique:** la tomodensitométrie puis l´imagerie par résonnance magnétique cérébrale retrouve une lésion extra-axiale intra-diploïque ptérionale gauche, avec une érosion de la structure osseuse (Figure 1), la lésion présentant un compartiment extracrânien de petite taille, et une composante intracrânienne volumineuse, hypointense en T1 et se rehausse très fortement après injection de gadolinium (Figure 2), spontanément hyperintense en T2 et FLAIR. On note également que l´artère méningée moyenne gauche traverse la masse dans une partie de son trajet confirmant le caractère hypervasculaire de la lésion, cette dernière semblait être intraosseuse, sans envahissement du revêtement piale ni du cortex adjacent, avec un discret effet de masse (Figure 3), tous ces éléments ont fait évoquer dans l´ordre un angiome intra-osseux, un hémangiopéricytome, ou un méningiome hémangioblastique.

**Figure 4:**
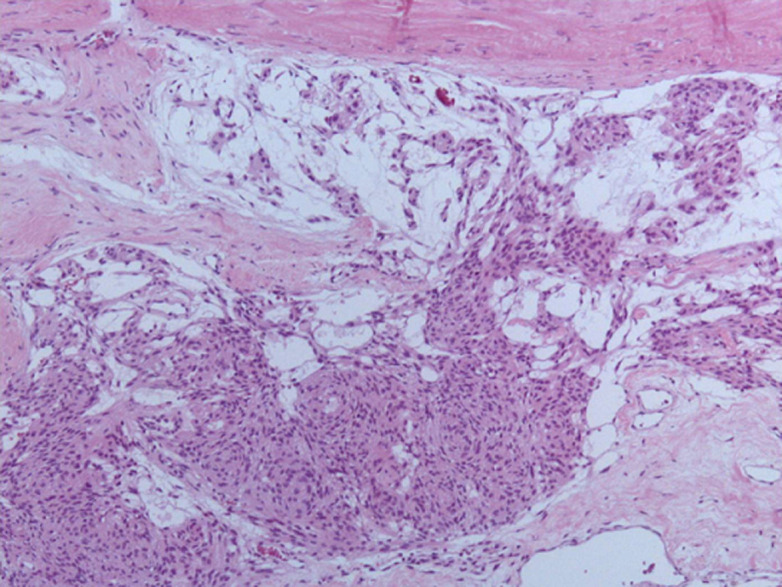
aspects histo-pathologiques: composante méningothéliale

**Figure 5:**
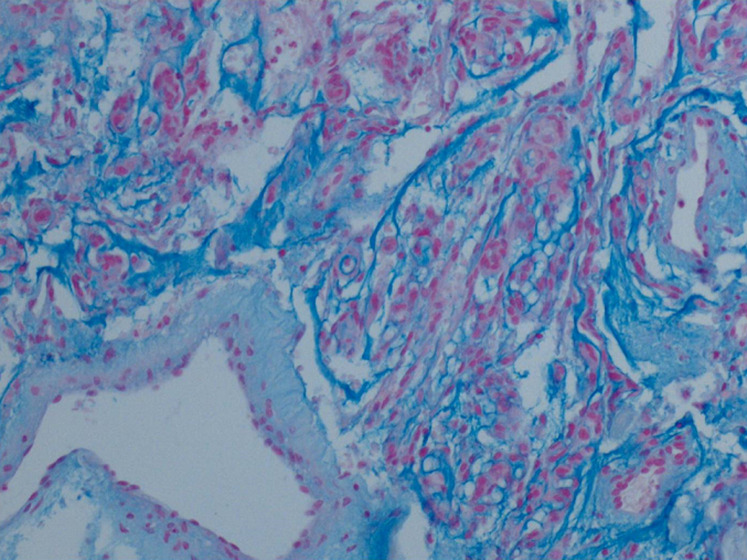
aspects histo-pathologiques: composante chordoïde

**Intervention thérapeutique:** une artériographie crânio-faciale a été réalisée avant l´intervention montrant une vascularisation presque exclusive par des branches issues de l´artère méningée moyenne homolatérale, permettant une embolisation des artères nourricières principales à l´aide de coils à détachement électromécanique, et dont le résultat final était la disparition de 80% du rehaussement anormal. L´intervention chirurgicale menée par voie ptérionale gauche, retrouve mise à part l´ostéolyse, une tumeur grisâtre gélatineuse et fibrineuse avec attache dure-mérienne obligeant à faire une incision circulaire à ce niveau, permettant une exérèse complète Simpson grade 1. L´examen anatomo-pathologique retrouve un tissu fibreux avec la présence de travées et de petits massifs de cellules physaliphores comportant un cytoplasme pâle éosinophile et des noyaux ovalaires sans atypie ni mitose manifeste, enchâssés dans une abondante substance mucoïde, infiltrant l´os et le muscle strié (Figure 4 et Figure 5).

**Figure 6:**
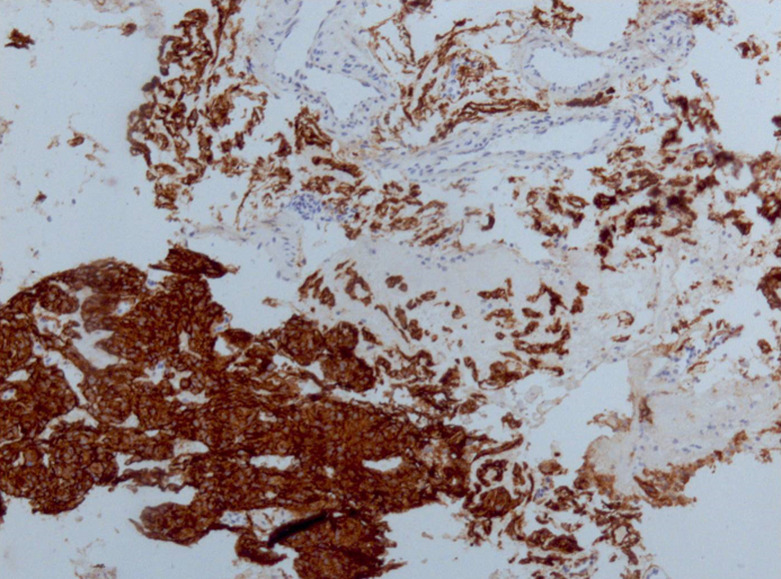
aspects histo-pathologiques: positivité de lEEMA

**Figure 7:**
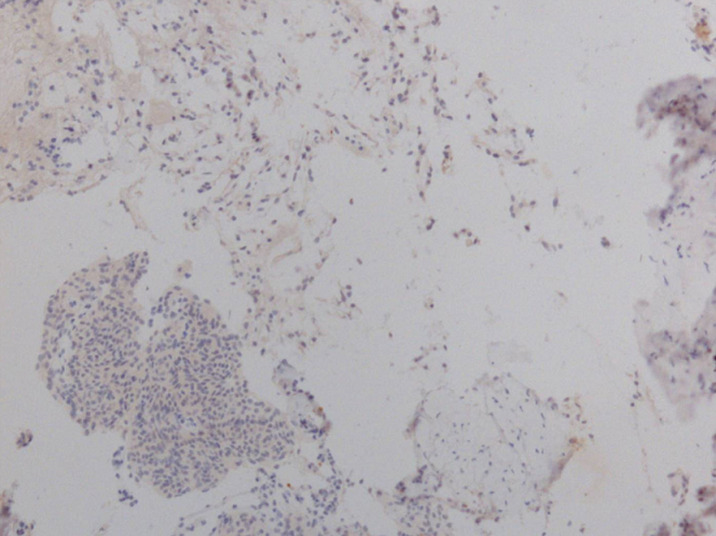
aspects histo-pathologiques: négativité de la cytokératine AE1/AE3

Par ailleurs, on note la présence de plusieurs petits amas de prolifération de cellules d´aspect méningothéliales, constitués de lobules avec enroulement des cellules. L´étude immunohistochimique (IHC) effectuée montre la positivité des cellules pour la vimentine et l´antigène épithélial membranaire (EMA) et la négativité pour la cytokératine. Il s´y associe une intense positivité pour les anticorps anti-récepteurs de la progestérone, mais sans marquage pour les anticorps anti-récepteurs de l´œstrogène. Enfin, l´index de prolifération Ki67 (clone MIB 1) est inférieur à 1%. Le diagnostic retenu est celui de méningiome chordoïde (Figure 6 et Figure 7).

**Suivi et résultats des interventions thérapeutiques:** un suivi régulier pendant trois ans, et la patiente présente un examen clinique normal et un bilan biologique sans particularité, notamment pas d´anémie à la numération formule sanguine. L´imagerie par résonnance magnétique (IRM) de contrôle montre une exérèse complète de la tumeur sans prise de contraste méningée ni œdème cérébrale.

**Perspective du patient:** la patiente est satisfaite de sa guérison, revue à la consultation il y a une année.

**Consentement du patient:** la patiente a accepté la publication anonyme de son expérience.

## Discussion

Les méningiomes chordoïdes ont été décrits pour la première fois par Kepes *et al*. en 1988, comme étant une tumeur qui se présente préférentiellement chez les enfants, et souvent associée à la maladie de Castelman [1]. Depuis notre compréhension de cette tumeur a considérablement augmentée, l´incidence estimée est de 0,5 à 1% des méningiomes traités chirurgicalement avec une légère prédominance féminine [4].

Les symptômes révélateurs diffèrent selon la localisation et la taille mais le symptôme le plus fréquent reste les céphalées, sans aucun signe spécifique objectif retrouvé dans la littérature des méningiomes chordoïdes [1,2,4,6]. La maladie de Castleman ou l´hyperplasie ganglionnaire angiofolliculaire, décrite pour la première fois en 1954 par Benjamin Castleman [7], est une maladie lymphoproliférative rare, typiquement sous forme des masses médiastinales ou hilaires hypervasculaires. Elle se révèle soit par un syndrome tumoral lié à la compression d´organes de voisinage, soit par un syndrome inflammatoire systémique avec hyperthermie, sueurs nocturnes et altération de l´état général, associé à une hypergammaglobulinémie. Kepes *et al*. [1] décrit sept jeunes patients présentant un méningiome chordoïde associé à quelques signes de la maladie de Castelman, notamment deux patients dont l´anémie hypochrome microcytaire, résolutive immédiatement après l´exérèse macroscopiquement complète de la tumeur, est réapparue avec la récidive locale de cette tumeur. Cependant, Couce *et al*. [4] ne rapporte aucun cas d´anémie ou dysglobulinémie parmi les 42 cas de méningiomes chordoïdes chez l´adulte; Hong-da *et al*. [8] a colligé 17 cas de méningiomes chordoïdes et dont 3 patients présentaient une anémie hypochrome microcytaire sans autre signe associé, notre cas présente également le même tableau. D'autres auteurs [4-6] ont constaté que l´infiltration lymphoplasmocytaire péritumorale d´un méningiome chordoïde est prédominé par les lymphocytes B chez les jeunes patients alors que chez les patients adultes, il existe une prédominance des lymphocytes T. Kaloshi *et al*. [9] pour sa part pense que l´expression tumorale de l´interleukin 6 pourrait expliquer les manifestations systémiques de la maladie de Castelman, et que la disparition des symptômes systémiques après une exérèse complète de la tumeur appuie son hypothèse.

La confirmation diagnostic du méningiome chordoïde est histopathologique, il s´agit d´une prolifération de cellules tumorales épithéloïdes ou fusiforme agencée en travées et cordons, comportant un cytoplasme pâle et éosinophile avec des noyaux ovalaire et dense, baignant dans une trame mucoïde abondante, avec expression à l´immunohistochimie de l´EMA et la vimentine, et négativité de la cytokératine ce qui la différentie du chordome: le diagnostic différentiel principal à côté du gliome chordoïde qui exprime de façon intense la *glial fibrillary acid protein* (GFAP) [10]. Il existe d´autres diagnostics différentiels: ce sont des tumeurs avec cytoplasme pâle, une structure mucoïde/chordoïde et qui peuvent se développer dans et/ou proche du système nerveux central tels que: le chordome chordoïdes, chondrosarcome myxoïde, chondrosarcome de bas grade, enchondrome, carcinome métastatique mucineux et le carcinome métastatique de cellules rénales [3] (Tableau 1).

**Tableau 1:** informations générales

	Informations générales				Localisation tumorale		Manifestations systémiques		Pourcentage des éléments chordoïdes		IHC	Suivi	
	Nombre de cas	Les extrêmes de l'âge (l'âge moyen)	Nombre d'enfants moins de 18 ans	Sex ratio	Supra-tentorielle	Infra-tentorielle	Typique	Atypique	> 50 %	< 50 %	MIB-1 LI	radiothérapie	Taux de récidive
Yang *et al*. [10]	60	17-80 (41,4)	-	1:1,5	36,7%	63,3%	0%	100%	43,3%	56,6%	-	31,6%	28,3%
Epari *et al*. [2]	12	12-67 (32,4)	3 (25%)	1:1,4	66,7%	33,3%	100%	0%	91,6%	8,3%	1-2%	-	0%
Lin *et al*. [3]	11	43-77 (60,8)	-	1,2:1	91%	9 %	-	100%	69%	31%	0,3-25,8%	27%	20%
Kepes *et al*. [1]	7	8-19 (11,9)	6 (85,7%)	1:1,3	71,4%	28,6%	100%	-	-	-	-		28,6%
Wang *et al*. [6]	30	16-74 (49,6)	1 (5,8%)	1:1,3	80%	20%	0%	100%	69%	31%	1-10%	30%	19%
Couce *et al*. [4]	42	12-77 (47,4)	2 (5,2 %)	1:1,1	88%	12%	88%	12%	81%	19%	0,4-11,4% (5,2%)	-	42%

L´exérèse chirurgicale reste le traitement de choix et le résultat désiré sera une exérèse macroscopiquement totale, vu le haut potentiel de récidive de ce type de méningiome le classant au grade 2 de la classification de l’Organisation mondiale de la Santé (OMS), malgré l´absence des critères de malignité (index mitotique élevé, nécrose, envahissement cérébral), sauf que, dans des cas de méningiomes, tels que ceux envahissant le sinus caverneux, la région petroclival, la partie postérieure du sinus sagittale supérieure, ou le nerf optique, l´exérèse totale est impossible vu le risque vital ou par rapport aux structures vasculaires proches de la tumeur, dans ce genre de situations une exérèse subtotale suivie d´une radiothérapie adjuvante ou une radiochirugie stéréotactique est recommandée [3].

## Conclusion

Le méningiome chordoïde est une tumeur rare et particulière, même si les différentes séries ne trouvent pas toujours une association avec la maladie de Castelman, ni une préférence d´âge. C´est une tumeur redoutable de récidive. Le diagnostic est basé sur l´étude immunohistochimique et le traitement de choix est l´exérèse totale.
